# Development of a New Formulation Based on In Situ Photopolymerized Polymer for the Treatment of Spinal Cord Injury

**DOI:** 10.3390/polym13244274

**Published:** 2021-12-07

**Authors:** Gabrielle B. Novais, Stefane dos Santos, Robertta J. R. Santana, Rose N. P. Filho, John L. S. Cunha, Bruno S. Lima, Adriano A. S. Araújo, Patricia Severino, Ricardo L. C. Albuquerque Júnior, Juliana C. Cardoso, Eliana B. Souto, Margarete Z. Gomes

**Affiliations:** 1 Health and Environment Postgraduate Program, Tiradentes University, Aracaju 49032 490, Sergipe, Brazil; gabibarrozonovais@gmail.com; 2 Biomedicine Graduation Program, Tiradentes University, Aracaju 49032 490, Sergipe, Brazil; stefanesantos5294@gmail.com; 3 School of Pharmacy, Tiradentes University, Aracaju 49032 490, Sergipe, Brazil; robertta.jussara@souunit.com.br; 4 Laboratory of Morphology and Experimental Pathology (LMBE), Institute of Technology and Research (ITP), Tiradentes University, Aracaju 49032 490, Sergipe, Brazil; rose_nely@itp.org.br (R.N.P.F.); ricardo.patologia@uol.com.br (R.L.C.A.J.); margarete_zanardo@itp.org.br (M.Z.G.); 5 Department of Odontology, Paraiba State University, Campina Grande 58429 500, Paraíba, Brazil; lennonrrr@gmail.com; 6 Department of Pharmaceutical Sciences, Federal University of Sergipe, São Cristóvão 49000 100, Sergipe, Brazil; bruunoo_ita@hotmail.com (B.S.L.); adriasa2001@yahoo.com.br (A.A.S.A.); 7 Nanomedicine and Nanotechnology Laboratory (LNMed), Institute of Technology and Research (ITP), Av. Murilo Dantas, 300, Aracaju 49010 390, Sergipe, Brazil; patricia_severino@itp.org.br; 8 Laboratory of Biomaterials (LBmat), Institute of Technology and Research (ITP), Av. Murilo Dantas, 300, Aracaju 49010 390, Sergipe, Brazil; 9 CEB-Centre of Biological Engineering, Campus de Gualtar, University of Minho, 4710-057 Braga, Portugal

**Keywords:** methacryloyl gelatin, red propolis, spinal cord injury, flavonoids, formononetin

## Abstract

Spinal Cord Injury (SCI) promotes a cascade of inflammatory events that are responsible for neuronal death and glial scar formation at the site of the injury, hindering tissue neuroregeneration. Among the main approaches for the treatment of SCI, the use of biomaterials, especially gelatin methacryloyl (GelMA), has been proposed because it is biocompatible, has excellent mechanical properties, favoring cell adhesion and proliferation. In addition, it can act as a carrier of anti-inflammatory drugs, preventing the formation of glial scars. The present work presents the development and in situ application of a light-curing formulation based on GelMA containing a natural extract rich in anti-inflammatory, antioxidant and neuroprotective substances (hydroalcoholic extract of red propolis—HERP) in an experimental model of SCI in rats. The formulations were prepared and characterized by time of UV exposition, FTIR, swelling and degradation. The hydrogels containing 1 mg/mL of HERP were obtained by the exposure to UV radiation of 2 μL of the formulation for 60 s. The locomotor evaluation of the animals was performed by the scale (BBB) and demonstrated that after 3 and 7 days of the injury, the GelMA-HERP group (BBB = 5 and 7) presented greater recovery compared to the GelMA group (BBB = 4 and 5). Regarding the inflammatory process, using histomorphological techniques, there was an inflammation reduction in the groups treated with GelMA and GelMA-HERP, with decreases of cavitation in the injury site. Therefore, it is possible to conclude that the use of GelMA and GelMA-HERP hydrogel formulations is a promising strategy for the treatment of SCI when applied in situ, as soon as possible after the injury, improving the clinical and inflammatory conditions of the treated animals.

## 1. Introduction

Spinal cord injury (SCI) can be caused by trauma, disease or birth defect that may cause moderate or severe neurological sequels. SCI affects young adults between 15 and 29 years of age, which can cause irreparable damage or even death, in addition to being a public health problem, with a high investment for the public authorities, due to the long hospital stay that this type of condition requires [1]. After traumatic SCI, some structures are affected, such as the bone structure of the spine, musculature, blood-brain barrier (which protects the spinal cord) and nerve cells, such as neurons [2]. Thus, with neuronal damage, there is a temporary or permanent paralysis of the muscles of the autonomic nervous system and also of sensitivity depending on the location and extent of the injury [3].

SCI is characterized by two moments. The first is called a primary injury resulting from the trauma itself. The second moment is called a secondary lesion that refers to a cascade of progressive events divided into three stages, acute, subacute and chronic. The acute and subacute phases are characterized by ischemia, proapoptotic signaling and infiltration of inflammatory cells. These cells release pro-inflammatory cytokines and debris cytotoxic substances, such as DNA, ATP and reactive oxygen species, which lead to an increase in the microenvironment in the space affected by injury [2,4,5]. In the chronic phase, the axonal regeneration is avoided by the formation of an astroglial-fibrous scar formed in the cystic cavities through the process called reactive gliosis [1,4,6].

Currently, the treatment of SCI consists of reparative surgery [7], stem cell transplants [8], physical therapy [9] and pharmacotherapy [10]. Drug treatment is used to reduce the deleterious effects promoted in the second moment of SCI. Amongst the most common drugs, the synthetic glucocorticoid methylprednisolone has been used to decrease lipid peroxidation and inflammatory response. However, methylprednisolone is associated with serious side effects, such as infections, hemorrhage, gastrointestinal diseases. In addition, there is no prevention of the glial scar formation, which compromises local axonal regeneration [11]. As no effective therapies exist that can inhibit the side effects of the currently available drugs or the formation of the glial scar in order to reestablish nerve conduction, the search for new strategies for SCI treatment is necessary.

Pre-clinical studies using natural products by oral administration have been carried out, aiming at the discovery of new drugs, thus becoming a promising approach for the treatment of SCI. Among natural products, one of great interest is red propolis, which contains molecules, such as flavonoids, in its chemical composition, with several activities, among which anti-inflammatory [12,13,14,15] and neuroprotective [3,16] activities stand out. These pharmacological effects are extremely relevant to modulate the inflammatory response that occurs at the site of injury. Some studies using flavonoids for the treatment of SCI have shown positive results. According to Shi et al. (2016), naringenin provided a protective effect on SCI by regulating neuroinflammation [17]. This flavonoid was even formulated in multi-walled carbon nanotubes and was found to reduce the cytotoxicity on non-malignant cells (human fibroblasts) than free naringenin, with an improved anticancer effect on malignant lung cells (A549) [18]. In the study by Wang et al. (2021) [19], in addition to acting on the regulation of neuroinflammation and oxidative stress, the naringenin also acted on locomotor and tissue improvement by promoting the expression of the SDF-1a gene and protein via the SDF-1/CXCR4 signaling pathway in bone marrow-derived mesenchymal stem cells. Administering the drug in situ could act on axonal regeneration, whereas this repair does not take place upon its oral administration.

Among the alternatives, studies indicate effectiveness in the use of biomaterials that are materials of synthetic or natural origin that can be implanted at the injury site, capable of replacing or repairing tissues, in addition to being favorable for the placement of substances in situ [20]. Biomaterials include gelatin methacryloyl hydrogel (GelMA), a natural polymer based on collagen, which presents important characteristics, such as biocompatibility with biological tissue, biodegradability, bioreabsorption capacity, atoxicity, shares characteristics similar to the CNS, presents a matrix similar to the CNS with high water content that favors cell growth, adhesion capacity, proliferation and cell differentiation [3,21]. This polymer also serves as a framework at the site of the lesion, where cystic cavities are formed, being able to join the ends of the spinal cord, in addition to acting in the controlled delivery of a drug [22].

Some studies have proven the effect of implanting hydrogels in situ. Fan et al. (2018) used a combination of GelMA associated with neural stem cells derived from pluripotent stem cells and achieved axonal regeneration by inhibiting the glial scar [3]. Wang et al. (2017) used a thermosensitive heparin-poloxamer hydrogel loaded with an acidic fibroblast growth factor (aFGF) that promoted neural and axonal rehabilitation, inhibition of glial scarring, suppression of the inflammatory response and motor recovery [23].

This work describes the development of a new formulation based on GelMA containing hydroethanolic extract of red propolis (HERP), to be applied in situ in a murine model of SCI, in order to inhibit the formation of glial scar at the site of injury, as well as the cascade of secondary events from the anti-inflammatory potential of HERP.

## 2. Materials and Methods

### 2.1. Materials

Methacrylic anhydride, collagenase type II, gelatin methacryloyl type A, irgacure 2959, dialysis membrane, chromatographic standards (formononetin, daidzein, liquiritigenin and Biochanin A) and Phosphate Buffer Solution (PBS—7.4) were purchased from Sigma-Aldrich^®^ (St. Louis, MO, USA) and red propolis was purchased from Unipropolis (Alagoas, Brazil).

### 2.2. Preparation of Extract

Red propolis from Alagoas/Brazil was provided from Unipropolis latitude (9°68′50.3″ S) and longitude (35°76′67.7″ W) with an elevation of 5 m. The sample was stored and maintained at −20 °C. The sample was added to ethanol 70% in a proportion of 1:12.5 *w*/*v*). The suspension was submitted to extraction in an ultrasound bath (Ultra Cleaner 1400A, Unique, Indaiatuba, São Paulo, Brazil) for 1 h at 25 °C. Subsequently, it was centrifuged (1800× *g* for 15 min), and the supernatant was dried in a fume hood stored under refrigeration, without exposure to light. The yield of the extracted material was calculated based on the percentage of dry mass, taking the initial mass of propolis before extraction and the material recovered after extraction and washing of the solvent as reference [24].

### 2.3. High-Performance Liquid Chromatography (HPLC)

The red propolis extract was diluted with methanol/water (50:50 *v*/*v*) to a concentration of 400 µg/mL (10 mg of red propolis in 25 mL of methanol/water (milli-Q system), and the sample was submitted to an ultrasonic bath for 45 min. Before HPLC injection, the sample was filtered with a membrane filter (PTFE—0.45 μm). HPLC analysis was performed using a high-performance liquid chromatography system that consisted of a degasser DGU-20A3, two LC-20AD pumps, a 20A HT automatic injector, a CTO-20A column oven, a SPDM20Avp photodiode array detector (DAD) and a CBM-20A system controller (Shimadzu Co., Kyoto, Japan). Chromatography was performed using the analytical column C18 Phenomenex Luna^®^ 4.6 × 150 mm (particle size of 5 µm). The sample injection volume was 20 µL, and the flow rate of the mobile phase was 1.0 mL/min. The solvents used in the mobile phase were: (A) 1% acetic acid (Neon, São Paulo, Brazil) in water (Milli-Q system, Millipore, Bedford, MA, USA) and (B) methanol (HPLC Panreac, Darmstadt, Germany). The elution profile was a linear gradient elution starting with 40% B over 10 min; 45–50% B for 10–15 min; 50–55% B for 15–20 min; 55% B for 20–35 min; 55–100% B for 35–50 min; 100–40% B for 50–60 min; returning to the initial conditions and finishing the analysis. The detector was set at 280 nm to acquire the chromatogram. The compounds were identified through standard co-injections comparing retention times and ultraviolet absorption spectra. Biochanin A, daidzein, formononetin and liquiritigenin (C_16_H_12_O_5_) were obtained from Sigma-Aldrich^®^ and diluted with methanol to a concentration of 100 µg/mL (stock solution). For quantitative analysis, the calibration curves for each standard were prepared in five different concentrations: 2, 4, 6, 8 and 10 µg/mL. The data were obtained using the LC Solution software [25].

### 2.4. Methacrylated Gelatin Synthesis

Gelatin methacryloyl was synthesized as previously described by de Vasconcelos et al. (2020) [26]. Type A porcine skin gelatin 10% (*w*/*v*) was dissolved in phosphate buffer solution (pH 7.4, 60 °C). After one hour, methacrylic anhydride (1% *v*/*v*) was dropped into the solution, and the reaction occurred at 50 °C for 3 h under constant stirring. The mixture was homogenized for 3 h at 50 °C, and to stop the reaction, the mixture was dialyzed with distillate water that was changed every 2 h for 5 days to remove salts and methacrylic acid. The solution was lyophilized for 5 days to generate a white, porous foam and was then stored at −80 °C until later use.

### 2.5. Development of the GelMA Hydrogel with and without Extract

**Figure 1:**
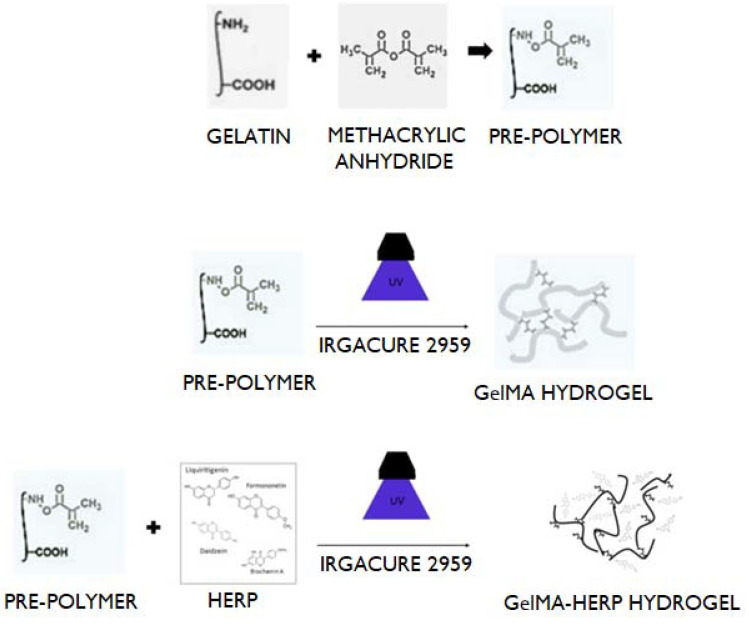
Preparation of the hydrogels. Reaction to prepare the prepolymer (**top**), photopolymerization step to obtain GelMA hydrogel (**middle**) and photopolymerization step to obtain GelMA-HERP hydrogel (**bottom**).

The GelMA hydrogel was prepared as described previously [27]. To prepare a solution, 1 mL of phosphate buffer (pH 7.4) was added to 0.05 g of the photoinitiator 2-hydroxy-1-(4-(hydroxyethoxy)phenyl)-2-methyl-1-propanone; Irgacure 2959, CIBA Chemicals, Basel, Switzerland) at 80 °C. Afterward, the lyophilized GelMA macromers were added to a concentration of 0.1 g and stirred until complete dilution. To obtain the GelMA hydrogel containing HERP, the previous formulation was homogenized with a previously prepared solution of HERP. This HERP solution was obtained by dissolving 0.01 g of HERP in 50 mL of 70% alcohol. To obtain the hydrogels, a photopolymerization process was carried out with ultraviolet light (6.7 mW.Cm^2^, 360–480 nm) for different volumes and periods of exposure. Figure 1 depicts the synthesis of the hydrogels.

### 2.6. Hydrogel Swelling Analysis

For the swelling test, hydrogels (2 µL) with or without HERP were photopolymerized by exposure to ultraviolet light for 60 and 90 s. The samples were weighed and added to PBS pH 7.4 at 37 °C for 24 h; the discs were removed and weighed again. In this way, the percentage of swelling (% I) was calculated by the ratio between the mass of absorbed buffer (m–m0) and the mass of the hydrogel before swelling (m0). Ten replicates were performed [26].

### 2.7. GelMA Biodegradation

The hydrogel samples (2 µL) after light-curing for 60 or 90 s were weighed and placed in 1 mL of phosphate buffer solution containing 2 U/mL of type II collagenase at 37 °C for 24 h. After this period, the collagenase solution was eliminated. The hydrogel was weighed again. The percentage degradation calculation was equivalent to the ratio between the mass after digestion and the initial mass of the samples. Ten replicates were performed [28].

### 2.8. Fourier Transform Infrared Spectroscopy

Fourier Transform Infrared Spectroscopy (FT-IR) analysis of GelMA hydrogel chemical composition was performed using Fourier Transform Infrared Spectroscopy with Attenuated Total Reflectance (FTIR-ATR), according to Aldana et al. (2019), with modifications, using Cary 630 FTIR Spectrometer Agilent Technologies, Inc.© (Santa Clara, CA, USA) equipped with a zinc select crystal (ZnSe). The wavelength used for the recording of spectra was in the range of 4000–600 cm^−1^.

### 2.9. In Vivo Experiments

#### 2.9.1. Experimental Groups and Spinal Cord Injury Surgery

Female *Wistar* rats (*Rattus norvegicus Albinus*) (150–250 g n = 48) were obtained from the Tiradentes University nursery. The care and use of all animals were in accordance with the guidelines established by CEUA. All rats were housed under controlled environmental conditions. The animals were randomized and divided into four groups containing 12 rats each: Laminectomy (without spinal cord involvement), Lesion (bilateral hemisection with the addition of 2 µL of PBS in each hemibody by epidural route), Lesion-GelMA (bilateral hemisection with the addition of 2 µL Epidural GelMA) and GelMA-HERP lesion (bilateral hemisection with the addition of 2 µL of GelMA-HERP in each hemibody via epidural). After seven days, the animals were sacrificed and submitted for analysis, with 24 rats for histomorphological evaluation and 24 for antioxidant testing. To induce spinal cord injury (SCI), the animals were weighed and anesthetized by injecting a mixture of 100 mg/kg of 10% ketamine and 14 mg/kg of 2% xylazine for each 100 g of the animal intraperitoneally. After interrupting the corneal reflexes, trichotomy was performed, followed by dorsal asepsis with iodine and 70% ethanol. Subsequently, laminectomy was performed on the T9 and T10 vertebrae after spine exposure, followed by bilateral hemisection for the injured groups. After hemisection, 2 µL of the formulations were added to each hemibody and photopolymerized by UV Light 6.7 mW cm2 (360–480 nm) for 60 s at room temperature. After the respective treatment, the paravertebral muscles and the skin were closed in layers and disinfected with 70% ethanol again. The rats were submitted to prophylactic antibiotic therapy with (Pentabiol) and added to heated cages, with access to food and water, in addition to receiving massages three times a day for three days to restore urination and desertion [3,26,29]. The experimental animal handling protocols and procedures were approved by the Animal Use Ethics Committee of Universidade Tiradentes.

#### 2.9.2. Assessment of Recovery of Locomotion

The locomotion recovery analyzes were carried out by Basso–Beattie–Bresnahan (BBB) locomotion scale, performed on days 0, 1, 3 and 7. In summary, BBB scores range from 0 (no movement) to 21 (full function) [23,30].

#### 2.9.3. Histological Analysis

The rats were sacrificed after 7 weeks, and the bone marrow was dissected afterward; the tissues were removed and added to 10% formaldehyde for 5 days. After this process, the marrow was embedded in paraffin, and after making the slides, the sections were stained in Hematoxylin and eosin (H&E). The slides were analyzed by a microscope, where aspects of the inflammatory process were observed, such as hemorrhage, edema, hyperemia, infiltration of inflammatory cells, as well as a presence of CNS resident cells and cavitation in the lesion area [31].

### 2.10. Statistical Analysis

The data were submitted to Shapiro–Wilk’s normality analysis to verify the type of data distribution. When the results are presented with parametric distribution, they are subjected to analysis of variation (ANOVA) followed by Tukey’s post-test. No statistical difference was considered when *p* > 0.05.

## 3. Results and Discussion

**Figure 2:**
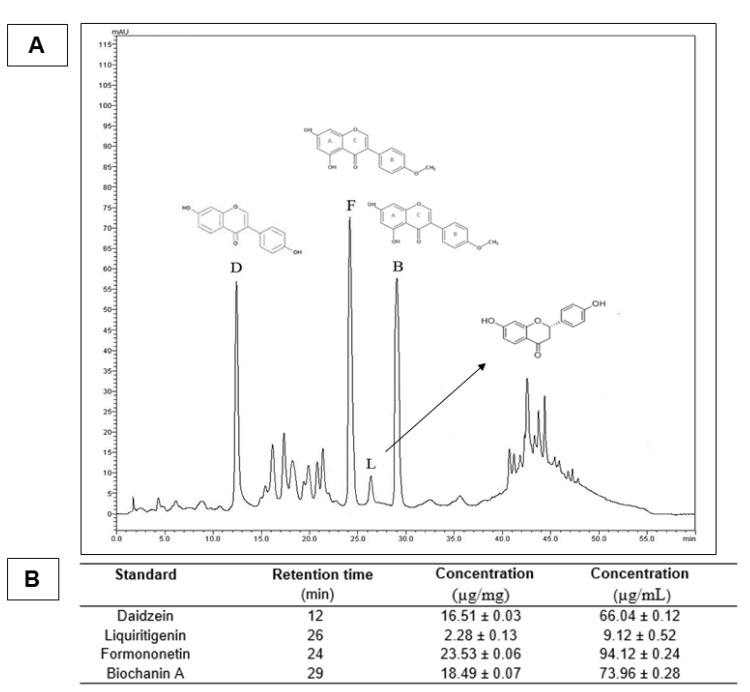
(**A**) Chromatographic profile of the hydroalcoholic extract of red propolis (HERP). Chemical markers; D: daidzein (retention time = 12 min); F: formononetin (retention time = 24 min); L: liquiritigenin (retention time = 26 min) and B: biochanin A (retention time = 29 min). (**B**) Quantification of the main chemical markers in HERP.

The yield of the red propolis extraction process was 51.2%, similar to what was found by Almeida et al. (2013) [32], who obtained 48.8%. The chromatogram of HERP showed four peaks corresponding to daidzein, formononetin, liquiritigenin and biochanin (Figure 2). Mendonça et al. (2015) studied the qualitative and quantitative seasonal variations of isoflavones in HERP [33]. The results showed a high variation of bioactive concentrations, but the chromatographic profile showed no huge change through the year, showing the formononetin as the main isoflavone. The formononetin presented a higher concentration of HERP (94.12 µg/mL), confirming it as the main bioactive maker, as observed in previous studies [34,35,36]. A high concentration of biochanin A in HERP (73.96 µg/mL) was also observed. These molecules have important antioxidant properties [35,37,38]. Formononetin and biochanin A were able to eliminate free radicals from the peroxidation process, decreasing the oxidative injury [39,40]. Salim (2017) suggested that several pathologies [41], such as SCI, can promote the excessive production of ROS, which leads to the involvement of tissues from trauma, causing cellular metabolic alteration, promoting an increase in radical-forming enzymes, aggravating the patient’s clinical condition by oxidative stress.

Formononetin, the main chemical marker of HERP, has anti-inflammatory activity and shows benefits to treat several disorders in in vivo studies [16,42]. The same performance has been observed for HERP studies. Almeida et al. (2013) showed an improvement in the healing process of wounds treated with biomembranes containing HERP [32]. The antinociceptive and anti-inflammatory activity of HERP and formononetin were described by Cavendish et al. (2015) [42], showing the decrease of edema, nociception induced by glutamate and inhibition of leukocytes migration. The anti-inflammatory and neuroprotective activities of HERP on nervous tissues were studied in a sciatic nerve model using rats. The authors showed functional recovery and axonal repair [16]. However, HERP has low solubility in an aqueous medium, and the incorporation of the extract in the hydrogel was a challenge. The incorporation of HERP in GelMA formulation needed previous HERP solubilization in a co-solvent (ethanol), and after this, the incorporation was possible. Another important point that must be defined is the volume of prepolymer and time of UV exposition. The ideal photopolymerization time to UV exposition is up to 60 s [27], because after this time, despite the polymerization process being favored, it can promote cellular death [43].

**Figure 3:**
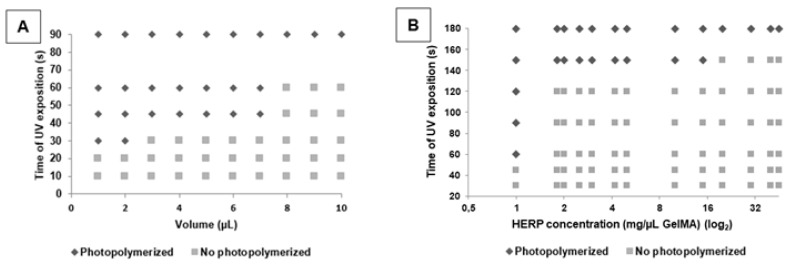
Influence of the formulation volume, time of UV exposure and HERP concentration on the photopolymerization of formulations: (**A**) GelMA; (**B**) GelMA-HERP (2 µL). Legend: Photopolymerized = Hydrogel formation; no photopolymerized = Insufficient time for light-curing the formulation.

To establish the ideal conditions of photopolymerization (UV exposition time and formulation volume), different volumes of GelMA formulation were submitted to increase the time of UV exposure. GelMA formulations were able to form hydrogels at 30 s and the maximal volume of 2 µL. When we used higher volumes of formulations, the time of UV exposition was also higher (Figure 3A). Vasconcelos et al. (2020) showed that 2 µL is enough to fill the injury site [26]. Therefore, 2 µL of formulation has been used and tested the incorporation of increasing concentrations of HERP, searching the ideal concentration to obtain the hydrogel.

Figure 3B presents the results of different concentrations of HERP incorporated in GelMA formulation (2 µL) submitted to increasing UV time exposure. After HERP incorporation (1 mg/µL), we needed more time to obtain the hydrogel (60 s). These results pointed to that the presence of isoflavones in formulation difficulted the photopolymerization reaction, probably to interfere in the interaction of gelatin methacryloyl chains. Therefore, we decided to prepare GelMA and GelMA-HERP hydrogels using a volume of 2 µL, 60 s UV exposure time and 1 mg/µL of HERP. This time is safe for neuronal cells and the volume enough to fill the lesion.

**Figure 4:**
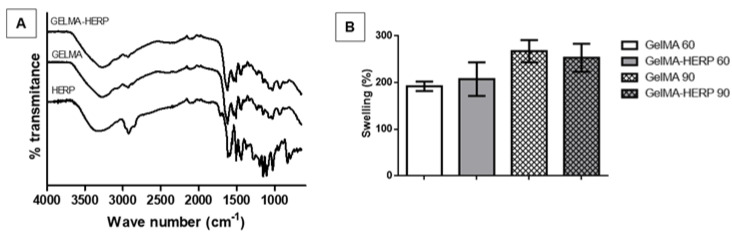
(**A**) FTIR spectra of lyophilized hydrogels of GelMA-HERP, GelMA and HERP in the range between 1000 and 1700 cm^−1^. (**B**) Swelling index (mean; bar as standard deviation) of the GelMA and GelMA-HERP hydrogels (1 mg/mL HERP; time of UV exposure 60 and 90 s). ANOVA followed by Tukey’s post-test.

In the FTIR results (Figure 4A), both hydrogels present similar spectral profiles compatible with protein molecules. They showed a broad band at 3274 cm^−1^ related to -OH groups in hydrogen bonds. At 1617 cm^−1^, we observed an intense band related to the amide I group (C=O stretching). A band at 1576 cm^−1^ refers to C-N-H flexion [44]. The spectra of GelMA and GelMA-HERP did not show differences. This result may be due to the low concentration of HERP used in the hydrogel (1 mg/mL) in relation to the concentration of gelatin methacryloyl (10 mg/mL). In addition, the molecules present in the HERP have a lower molar mass than that of the gelatin methacryloyl, which causes the vibrations of the bonds present in the gelatin to overlap those of the HERP molecules. Thus, the molecules present in the HERP are diluted in the formulation, with no relevant change in the GelMA-HERP spectrum; no interaction between the polymer and active substances was suggested. The results also suggested that the molecules from HERP did not affect the polymeric organization. Similar results were found by Rahali et al. (2017) [44], who observed that samples of hydrogels containing lipid nanoparticles in low concentrations did not disorganize the polymeric net.

The swelling of hydrogels obtained with 60 and 90 s of UV exposure was greater than 200% (Figure 4B). There was no significant difference in the swelling index of the hydrogel GelMA and GelMA-HERP, as well as the time of light-curing did not influence this property (*p* = 0.199965). To treat injuries, hydrogels must have limited levels of swelling, as this can further aggravate the injured site due to the compression that will occur at the site. It must also be considered that the light-curing time influences the swelling capacity, most likely due to the degree of crosslinking. However, in the present study, even with a high degree of swelling, the degradability of the hydrogel can occur simultaneously, preventing compression at the injury site. The incorporation of HERP in the hydrogel did not alter the degradation profile of the polymer. There was no significant difference between the degradability of the hydrogel GelMA (65.5% ± 1.1%) and GelMA-HERP (63.2% ± 2.1%) after 24 h of testing (*p* = 0.3436), similar to those found in the literature [45,46].

The degree of hydrogel swelling provides important information about the solute-solvent interaction and is relevant to assessing the degree of increase in the volume of the material after immersion in aqueous solvents. A high amount of swelling could aggravate the functional and inflammatory condition in SCI, as it causes compression in the area [47]. On the other hand, a lower swelling degree may indicate little interaction with the aqueous medium, disfavoring the HERP diffusion at the lesion site. A study carried out by Rahali et al. (2017) [44], demonstrated that the swelling of the gelatin methacryloyl hydrogel absorbed in PBS was four to five times the weight of the water, obtaining a swelling degree of around 200%.

The change in the degree of crosslinking (number of chemical interactions resulting from the photopolymerization reaction) can vary depending on the time of exposure to radiation and due to the presence of other substances in the formulation. These interactions alter the pore sizes of the hydrogels and the exposure of the hydrophilic sites of the gelatin molecule. However, the results did not show significant differences. The incorporation of HERP also did not affect the degree of swelling, suggesting that the molecules present in the extract did not affect the potential for water uptake by the hydrogel.

**Figure 5:**
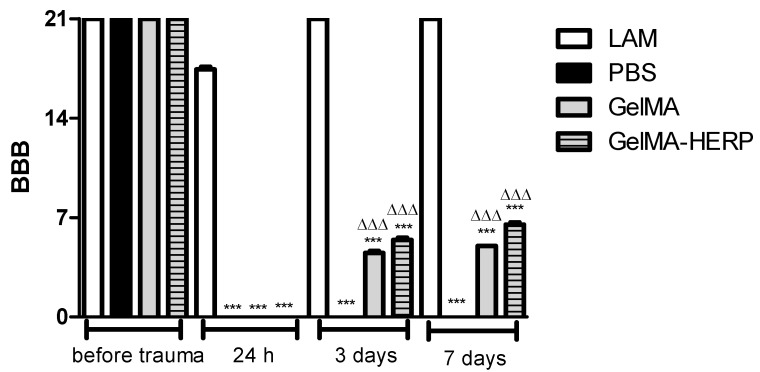
Functional assessment by the BBB scale of the LAM (laminectomy), PBS (vehicle), GelMA (gelatin methacryloyl hydrogel) and GelMA-HERP (gelatin methacryloyl hydrogel-containing hydroalcoholic extract of red propolis) groups. Before surgery, the groups showed no significant difference, presenting a BBB score of 21 in all groups. One day after the injury, the groups showed a significant difference when compared to the LAM group presenting (BBB = 0). After 3 and 7 days, the PBS group did not show functional improvement when compared to the LAM group, which showed a maximal score. The groups treated with GelMA and GelMA-HERP showed functional improvement, with GelMA-HERP showing the greatest recovery. ANOVA followed by Tukey’s post-test. The symbols on the columns identify the groups that present a significant difference in relation to the (***) LAM and (ΔΔΔ) PBS group for *p* < 0.001, at the same time after trauma.

For in vivo analysis, the formulations GelMA and GelMA-HERP were light-cured in situ in a model of bilateral hemisection of the spinal cord, as also performed by de Vasconcelos et al. [26] and Chedly et al. [29]. In the present study, a functional evaluation using the BBB scale was performed prior to surgery and 24 h, 3 and 7 days after the surgical procedure (Figure 5).

One day before surgery, all groups had preserved functional capacity (BBB = 21). However, after 24 h from the surgical procedure, only the LAM group showed significant functional improvement. These findings are important and demonstrate that the performance of laminectomy did not cause damage to the spinal cord. The animals in the vehicle group (PBS) showed zero as a result of BBB values, 1, 3 and 7 days after surgery. This result may be related, at least in part, to the damage generated to the spinal cord, which undergoes a sequence of pathological changes after injuries of traumatic origin, such as the appearance of edema, hemorrhage, necrosis of neuronal tissue, demyelination, formation of cysts and cavitation. All these alterations cause significant functional impairment [48]. These findings agreed with that of previous studies that evaluated the locomotor and histomorphological parameters of spinal cord trauma in a rodent model [49].

The groups treated with GelMA and GelMA-HERP showed improvement in locomotor function when compared to the PBS group, and the GelMA-HERP group showed higher BBB scores, statistically different from GelMA (*p* < 0.001). These results suggest that the application of the hydrogel-containing HERP may have promoted the reduction of the damage caused to the spinal cord. These results corroborate the morphological findings of the animals in this group, which evidenced a probable reduction in the inflammatory process and in the diameter of the cavitation. This finding is compatible with the results of Piantino et al. (2006) [50], who observed that the application of an injectable hydrogel promoted axonal regeneration and, consequently, improved the locomotor parameters of rats submitted to spinal cord injury. In addition, the bioactive compounds present in the HERP may play a fundamental role in spinal repair dynamics.

**Figure 6:**
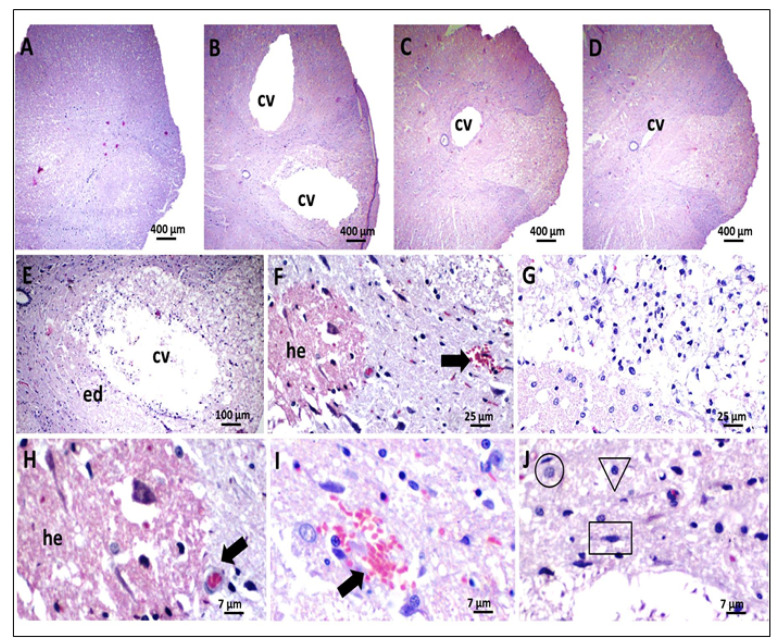
Photomicrographs of HE-stained histological sections representative of the experimental groups. (**A**) Group submitted only to laminectomy (LAM), presenting a well-preserved cytomorphological and architectural structure consistent with normality (40×). (**B**) Control group treated with phosphate buffer (PBS) with extensive cavitation in the middle of damaged spinal tissue and intense pericavitary edema (40×). (**C**,**D**) GelMA and GelMA-HERP groups, respectively, showing an intense reduction in tissue cavitation (40×). Details of the morphological changes observed in the PBS group, expressed by (**E**) pericavitary interstitial edema (100×), (**F**) extensive hemorrhagic areas and hyperemia (400×) and (**G**) possible poll of intracavitary inflammatory cells (400×). (**H**–**J**) enlarged magnification, highlighting hemorrhage, hyperemia and infiltration by glial cells (800×). Captions: cv—tissue cavitation; ed—interstitial edema; he—hemorrhagic areas; arrows—capillary hyperemia; circle—astrocyte; triangle—oligodendrocyte and rectangle—microglia.

In the histological sections of the spinal cord from the animals of the LAM group, we observed preservation of tissue architecture and the absence of any degenerative process (Figure 6). These findings are important and demonstrate that the performance of laminectomy did not cause morpho-architectural changes in the spinal cord, as already evidenced by the maximum scores of the motor assessments of the animals in this group. However, lesions and extensive cavitation were observed in the vehicle PBS group, in addition to hemorrhage, foci of necrosis and inflammation. These results were also observed by Kang et al. (2010) [51], indicating that the trauma model used in the present study was able to cause histological lesions correlated with severe motor deficits, such as those previously described. These findings are also compatible with that of previous studies that evaluated the effects of spinal trauma in a murine model [52].

In the morphological analysis of the spinal cord, the LAM group (submitted only to laminectomy), the animals presented medullar tissue with preservation of the tissue architecture and absence of alterations that would characterize any degenerative process. The spinal cord was observed with clear individualization of white and gray substances. The white substance occupied the most peripheral part of the spinal cord in all its extension, being constituted of axonal fibers, astrocytes, oligodendrocytes, microglia and support tissue with usual characteristics, besides discrete capillary blood vessels, sometimes hyperemic. Surrounded by white matter is gray matter, consisting of neuronal bodies, axonal fibers, astrocytes, oligodendrocytes, microglia and some blood vessels. An intact medullary or ependymal canal was also identified, located in the central region of the spinal cord, precisely in the central commissure of the gray matter (Figure 6A).

In the PBS group (subjected to injury and treated with vehicle), extensive cavitations were observed, affecting white and gray substances, intense pericavitary edema, moderate to severe axonal swelling and degeneration, areas of fibrinoid necrosis, hemorrhagic foci, occasional capillary vessels and inflammatory reaction. These were predominantly mononuclear, with possible neutrophil polymorphonuclear cells (Figure 6B,E–J).

In relation to animals in the GelMA group (subjected to injury and treated with hydrogel) and GelMA-HERP (subjected to injury and treated with hydrogel-containing HERP), similar histological patterns were verified with a slight improvement in the GelMA-HERP group. In both groups, there was a marked reduction in the diameter of tissue cavitation, slight multifocal axonal degeneration associated with axonal swelling and areas of necrosis quite scarce and/or absent. It was also found that the tissue architecture of the animals in the GelMA and GelMA-HERP groups was morphologically better than that observed in the PBS group. Occasional hyperemic capillary vessels, inflammatory cells and erythrocyte ingrowth have also been found relatively frequently (Figure 6C,D).

Many studies have evaluated the effect of applying hydrogels to spinal cord injury models to assess the neuronal repair process and have obtained the formation of microenvironments favorable to repair [50,51,52,53]. In the present study, the morphological analysis of the medullar tissue of the animals treated with GelMA and GelMA-HERP demonstrated a probable reduction in tissue cavitation compared to the PBS group, in addition to providing possible improvement from the morphological point of view of the medullary tissue architecture. This finding was compatible with a recent study, which showed that the administration of a thermosensitive hydrogel provided an almost complete reduction of cavitation and cystic spaces in an experimental model, in addition to providing greater interaction of the biopolymer with the resident macrophages, which are activated to produce MMP-9, providing the remodeling of the glial extracellular matrix [54].

It is suggested that the lesions, apparently more discrete in the GelMA and GelMA-HERP group, probably result from the dynamic interaction of the biopolymer with cellular components and/or interstitial matrix in the host tissue, providing physical support and structural stabilization, and thus, creating a favorable microenvironment for the repair process to occur [54].

## 4. Conclusions

From this work, we conclude that the hydroethanolic extract of red propolis (HERP), containing formononetin as a major compound, carried in GelMA hydrogels (GelMA-HERP) was light-cured with a volume of 2 µL in vitro and in situ, with an exposure time to UV light of 60 s, with potential use for in situ applications. The biocompatible and tunable properties of the biopolymer promote the opportunity for its use in cell cultures and in tissue engineering by the interaction with cellular components and interstitial matrix. In this study, motor functions and histomorphological changes were improved with in situ treatment using GelMA and GelMA-HERP, demonstrating that the formulation is promising to be used in SCI.

## Data Availability

Data are available from corresponding authors upon request.

## References

[1] Ahuja C.S., Wilson J.R., Nori S., Kotter M.R.N., Druschel C., Curt A., Fehlings M.G. (2017). Traumatic spinal cord injury. Nat. Rev. Dis. Primers.

[2] He Z., Zang H., Zhu L., Huang K., Yi T., Zhang S., Cheng S. (2019). An anti-inflammatory peptide and brain-derived neurotrophic factor-modified hyaluronan-methylcellulose hydrogel promotes nerve regeneration in rats with spinal cord injury. Int. J. Nanomed..

[3] Fan L., Liu C., Chen X., Zou Y., Zhou Z., Lin C., Tan G., Zhou L., Ning C., Wang Q. (2018). Directing Induced Pluripotent Stem Cell Derived Neural Stem Cell Fate with a Three-Dimensional Biomimetic Hydrogel for Spinal Cord Injury Repair. ACS Appl. Mater. Interfaces.

[4] Ahuja C.S., Nori S., Tetreault L., Wilson J., Kwon B., Harrop J., Choi D., Fehlings M.G. (2017). Traumatic Spinal Cord Injury-Repair and Regeneration. Neurosurgery.

[5] Sámano C., Nistri A. (2019). Mechanism of Neuroprotection against Experimental Spinal Cord Injury by Riluzole or Methylprednisolone. Neurochem. Res..

[6] Venkatesh K., Ghosh S.K., Mullick M., Manivasagam G., Sen D. (2019). Spinal cord injury: Pathophysiology, treatment strategies, associated challenges, and future implications. Cell Tissue Res..

[7] Ojo O.A., Poluyi E.O., Owolabi B.S., Kanu O.O., Popoola M.O. (2017). Surgical decompression for traumatic spinal cord injury in a tertiary center. Niger. J. Clin. Pract..

[8] Lu Y., Gao H., Zhang M., Chen B., Yang H. (2017). Glial Cell Line-Derived Neurotrophic Factor-Transfected Placenta-Derived Versus Bone Marrow-Derived Mesenchymal Cells for Treating Spinal Cord Injury. Med. Sci. Monit..

[9] Ryu Y., Ogata T., Nagao M., Sawada Y., Nishimura R., Fujita N. (2018). Effects of Treadmill Training Combined with Serotonergic Interventions on Spasticity after Contusive Spinal Cord Injury. J. Neurotrauma.

[10] Rouanet C., Reges D., Rocha E., Gagliardi V., Silva G.S. (2017). Traumatic spinal cord injury: Current concepts and treatment update. Arq. Neuro-Psiquiatr..

[11] Mbori N.J., Chuan X.Y., Feng Q.X., Alizada M., Zhan J. (2016). Evaluation of the Combination of Methylprednisolone and Tranilast after Spinal Cord Injury in Rat Models. J. Korean Neurosurg. Soc..

[12] de Carvalho F.M.A., Schneider J.K., de Jesus C.V.F., de Andrade L.N., Amaral R.G., David J.M., Krause L.C., Severino P., Soares C.M.F., Bastos E.C. (2020). Brazilian Red Propolis: Extracts Production, Physicochemical Characterization, and Cytotoxicity Profile for Antitumor Activity. Biomolecules.

[13] Loureiro K.C., Barbosa T.C., Nery M., Chaud M.V., da Silva C.F., Andrade L.N., Correa C.B., Jaguer A., Padilha F.F., Cardoso J.C. (2020). Antibacterial activity of chitosan/collagen membranes containing red propolis extract. Pharmazie.

[14] Picolotto A., Pergher D., Pereira G.P., Machado K.G., da Silva Barud H., Roesch-Ely M., Gonzalez M.H., Tasso L., Figueiredo J.G., Moura S. (2019). Bacterial cellulose membrane associated with red propolis as phytomodulator: Improved healing effects in experimental models of diabetes mellitus. Biomed. Pharmacother..

[15] Devequi-Nunes D., Machado B.A.S., Barreto G.A., Rebouças Silva J., da Silva D.F., da Rocha J.L.C., Brandão H.N., Borges V.M., Umsza-Guez M.A. (2018). Chemical characterization and biological activity of six different extracts of propolis through conventional methods and supercritical extraction. PLoS ONE.

[16] Barbosa R.A., Nunes T.L., Nunes T.L., da Paixão A.O., Belo Neto R., Moura S., Albuquerque Junior R.L., Cândido E.A., Padilha F.F., Quintans-Júnior L.J. (2016). Hydroalcoholic extract of red propolis promotes functional recovery and axon repair after sciatic nerve injury in rats. Pharm. Biol..

[17] Shi L.B., Tang P.F., Zhang W., Zhao Y.P., Zhang L.C., Zhang H. (2016). Naringenin inhibits spinal cord injury-induced activation of neutrophils through miR-223. Gene.

[18] Morais R.P., Novais G.B., Sangenito L.S., Santos A.L.S., Priefer R., Morsink M., Mendonca M.C., Souto E.B., Severino P., Cardoso J.C. (2020). Naringenin-Functionalized Multi-Walled Carbon Nanotubes: A Potential Approach for Site-Specific Remote-Controlled Anticancer Delivery for the Treatment of Lung Cancer Cells. Int. J. Mol. Sci..

[19] Wang Y., Bai S., Cheng Q., Zeng Y., Xu X., Guan G. (2021). Naringenin promotes SDF-1/CXCR4 signaling pathway in BMSCs osteogenic differentiation. Folia Histochem. Cytobiol..

[20] Boisserand L.S., Kodama T., Papassin J., Auzely R., Moisan A., Rome C., Detante O. (2016). Biomaterial Applications in Cell-Based Therapy in Experimental Stroke. Stem Cells Int..

[21] Liu W., Zhong Z., Hu N., Zhou Y., Maggio L., Miri A.K., Fragasso A., Jin X., Khademhosseini A., Zhang Y.S. (2018). Coaxial extrusion bioprinting of 3D microfibrous constructs with cell-favorable gelatin methacryloyl microenvironments. Biofabrication.

[22] Dursun Usal T., Yucel D., Hasirci V. (2019). A novel GelMA-pHEMA hydrogel nerve guide for the treatment of peripheral nerve damages. Int. J. Biol. Macromol..

[23] Wang Q., He Y., Zhao Y., Xie H., Lin Q., He Z., Wang X., Li J., Zhang H., Wang C. (2017). A Thermosensitive Heparin-Poloxamer Hydrogel Bridges aFGF to Treat Spinal Cord Injury. ACS Appl. Mater. Interfaces.

[24] de Mélo Silva I.S., do Amorim Costa Gaspar L.M., Rocha A.M.O., da Costa L.P., Tada D.B., Franceschi E., Padilha F.F. (2020). Encapsulation of Red Propolis in Polymer Nanoparticles for the Destruction of Pathogenic Biofilms. AAPS PharmSciTech.

[25] da Silva R.O., Andrade V.M., Bullé Rêgo E.S., Azevedo Dória G.A., Santos Lima B.D., da Silva F.A., de Souza Araújo A.A., de Albuquerque Júnior R.L., Cordeiro Cardoso J., Zanardo Gomes M. (2015). Acute and sub-acute oral toxicity of Brazilian red propolis in rats. J. Ethnopharmacol..

[26] de Vasconcelos A.C.P., Morais R.P., Novais G.B., da Barroso S., Menezes L.R.O., dos Santos S., da Costa L.P., Correa C.B., Severino P., Gomes M.Z. (2020). In situ photocrosslinkable formulation of nanocomposites based on multi-walled carbon nanotubes and formononetin for potential application in spinal cord injury treatment. Nanomed. Nanotechnol. Biol. Med..

[27] Bertassoni L.E., Cardoso J.C., Manoharan V., Cristino A.L., Bhise N.S., Araujo W.A., Zorlutuna P., Vrana N.E., Ghaemmaghami A.M., Dokmeci M.R. (2014). Direct-write bioprinting of cell-laden methacrylated gelatin hydrogels. Biofabrication.

[28] Chen P., Xia C., Mei S., Wang J., Shan Z., Lin X., Fan S. (2016). Intra-articular delivery of sinomenium encapsulated by chitosan microspheres and photo-crosslinked GelMA hydrogel ameliorates osteoarthritis by effectively regulating autophagy. Biomaterials.

[29] Chedly J., Soares S., Montembault A., von Boxberg Y., Veron-Ravaille M., Mouffle C., Benassy M.N., Taxi J., David L., Nothias F. (2017). Physical chitosan microhydrogels as scaffolds for spinal cord injury restoration and axon regeneration. Biomaterials.

[30] Basso D.M., Beattie M.S., Bresnahan J.C. (1995). A sensitive and reliable locomotor rating scale for open field testing in rats. J. Neurotrauma.

[31] Pei J.P., Fan L.H., Nan K., Li J., Dang X.Q., Wang K.Z. (2017). HSYA alleviates secondary neuronal death through attenuating oxidative stress, inflammatory response, and neural apoptosis in SD rat spinal cord compression injury. J. Neuroinflamm..

[32] de Almeida E.B., Cordeiro Cardoso J., Karla de Lima A., de Oliveira N.L., de Pontes-Filho N.T., Oliveira Lima S., Leal Souza I.C., de Albuquerque-Júnior R.L. (2013). The incorporation of Brazilian propolis into collagen-based dressing films improves dermal burn healing. J. Ethnopharmacol..

[33] de Mendonca L.S., de Mendonca F.M.R., de Araujo Y.L.F.M., de Araujo E.D., Ramalho S.A., Narain N., Jain S., Orellana S.C., Padilha F.F., Cardoso J.C. (2015). Chemical markers and antifungal activity of red propolis from Sergipe, Brazil. Food Sci. Tech.-Brazil.

[34] de Moraes Porto I.C.C., De Almeida D.C.C., de Oliveira Costa G.V.C., Donato T.S.S., Nunes L.M., Do Nascimento T.G., dos Santos Oliveira J.M., Da Silva C.B., Dos Santos N.B., e Silva M.L.D.A. (2018). Mechanical and aesthetics compatibility of Brazilian red propolis micellar nanocomposite as a cavity cleaning agent. BMC Complementary Altern. Med..

[35] Batista C.M., Alves A.V.F., Queiroz L.A., Lima B.S., Filho R.N.P., Araújo A.A.S., de Albuquerque Júnior R.L.C., Cardoso J.C. (2018). The photoprotective and anti-inflammatory activity of red propolis extract in rats. J. Photochem. Photobiol. B Biol..

[36] Reis J.H.O., Barreto G.A., Cerqueira J.C., Anjos J.P.D., Andrade L.N., Padilha F.F., Druzian J.I., Machado B.A.S. (2019). Evaluation of the antioxidant profile and cytotoxic activity of red propolis extracts from different regions of northeastern Brazil obtained by conventional and ultrasound-assisted extraction. PLoS ONE.

[37] Andrade J.K.S., Denadai M., de Oliveira C.S., Nunes M.L., Narain N. (2017). Evaluation of bioactive compounds potential and antioxidant activity of brown, green and red propolis from Brazilian northeast region. Food Res. Int..

[38] Dantas Silva R.P., Machado B.A., Barreto G.A., Costa S.S., Andrade L.N., Amaral R.G., Carvalho A.A., Padilha F.F., Barbosa J.D., Umsza-Guez M.A. (2017). Antioxidant, antimicrobial, antiparasitic, and cytotoxic properties of various Brazilian propolis extracts. PLoS ONE.

[39] Frozza C.O., Garcia C.S., Gambato G., de Souza M.D., Salvador M., Moura S., Padilha F.F., Seixas F.K., Collares T., Borsuk S. (2013). Chemical characterization, antioxidant and cytotoxic activities of Brazilian red propolis. Food Chem. Toxicol. Int. J. Publ. Br. Ind. Biol. Res. Assoc..

[40] Righi A.A., Negri G., Salatino A. (2013). Comparative Chemistry of Propolis from Eight Brazilian Localities. Evid.-Based Complementary Altern. Med..

[41] Salim S. (2017). Oxidative Stress and the Central Nervous System. J. Pharmacol. Exp. Ther..

[42] Cavendish R.L., de Souza Santos J., Neto R.B., Paixão A.O., Oliveira J.V., de Araujo E.D., e Silva A.A.B., Thomazzi S.M., Cardoso J.C., Gomes M.Z. (2015). Antinociceptive and anti-inflammatory effects of Brazilian red propolis extract and formononetin in rodents. J. Ethnopharmacol..

[43] Occhetta P., Visone R., Russo L., Cipolla L., Moretti M., Rasponi M. (2015). VA-086 methacrylate gelatine photopolymerizable hydrogels: A parametric study for highly biocompatible 3D cell embedding. J. Biomed. Mater. Research. Part A.

[44] Rahali K., Ben Messaoud G., Kahn C.J.F., Sanchez-Gonzalez L., Kaci M., Cleymand F., Fleutot S., Linder M., Desobry S., Arab-Tehrany E. (2017). Synthesis and Characterization of Nanofunctionalized Gelatin Methacrylate Hydrogels. Int. J. Mol. Sci..

[45] Lai T.C., Yu J., Tsai W.B. (2016). Gelatin methacrylate/carboxybetaine methacrylate hydrogels with tunable crosslinking for controlled drug release. J. Mater. Chem. B.

[46] Nguyen A.H., McKinney J., Miller T., Bongiorno T., McDevitt T.C. (2015). Gelatin methacrylate microspheres for controlled growth factor release. Acta Biomater..

[47] Bae H., Ahari A.F., Shin H., Nichol J.W., Hutson C.B., Masaeli M., Kim S.-H., Aubin H., Yamanlar S., Khademhosseini A. (2011). Cell-laden microengineered pullulan methacrylate hydrogels promote cell proliferation and 3D cluster formation. Soft Matter.

[48] Ramadan W.S., Abdel-Hamid G.A., Al-Karim S., Abbas A.T. (2017). Histological, immunohistochemical and ultrastructural study of secondary compressed spinal cord injury in a rat model. Folia Histochem. Cytobiol..

[49] Zadeh-Ardabili P.M., Rad S.K., Rad S.K., Khazaài H., Sanusi J., Zadeh M.-a.-R.H. (2017). Palm vitamin E reduces locomotor dysfunction and morphological changes induced by spinal cord injury and protects against oxidative damage. Sci. Rep..

[50] Piantino J., Burdick J.A., Goldberg D., Langer R., Benowitz L.I. (2006). An injectable, biodegradable hydrogel for trophic factor delivery enhances axonal rewiring and improves performance after spinal cord injury. Exp. Neurol..

[51] Kang Y.M., Hwang D.H., Kim B.G., Go D.H., Park K.D. (2010). Thermosensitive polymer-based hydrogel mixed with the anti-inflammatory agent minocycline induces axonal regeneration in hemisected spinal cord. Macromol. Res..

[52] Jain A., Kim Y.T., McKeon R.J., Bellamkonda R.V. (2006). In situ gelling hydrogels for conformal repair of spinal cord defects, and local delivery of BDNF after spinal cord injury. Biomaterials.

[53] Tukmachev D., Forostyak S., Koci Z., Zaviskova K., Vackova I., Vyborny K., Sandvig I., Sandvig A., Medberry C.J., Badylak S.F. (2016). Injectable Extracellular Matrix Hydrogels as Scaffolds for Spinal Cord Injury Repair. Tissue Eng. Part A.

[54] Hong L.T.A., Kim Y.-M., Park H.H., Hwang D.H., Cui Y., Lee E.M., Yahn S., Lee J.K., Song S.-C., Kim B.G. (2017). An injectable hydrogel enhances tissue repair after spinal cord injury by promoting extracellular matrix remodeling. Nat. Commun..

